# Soy Products Ameliorate Obesity-Related Anthropometric Indicators in Overweight or Obese Asian and Non-Menopausal Women: A Meta-Analysis of Randomized Controlled Trials

**DOI:** 10.3390/nu11112790

**Published:** 2019-11-15

**Authors:** Yuze Mu, Tingyan Kou, Boyang Wei, Xuezhao Lu, Jingyao Liu, Huimin Tian, Wenwen Zhang, Bingkun Liu, Huihui Li, Wenbo Cui, Qiuzhen Wang

**Affiliations:** 1 Department of the College of Public Health, Qingdao University, Qingdao 266071, China; muyuze_2012@163.com (Y.M.); kid_conan@163.com (B.W.); luxuezhao@outlook.com (X.L.); liujingyao_2019@126.com (J.L.); tianhuimin_2019@126.com (H.T.); AgozvanCheung@126.com (W.Z.); lbk1613@163.com (B.L.); lihh980130@163.com (H.L.); c17863918284@163.com (W.C.); 2 Junan County Health Bureau, Linyi 276600, China; 13176851482@126.com

**Keywords:** soy products, weight, BMI, soy protein, isoflavones, soy fiber

## Abstract

Background: The effect of soy products on the weight of overweight or obese people is controversial, so we aimed to conduct a systematic review and a meta-analysis of published randomized controlled trials to analyze whether supplementation with soy products can help them to lose weight. Methods: The relevant data before January 2019 in PubMed, Embase and Cochrane Library were searched. A random-effect model was adopted to calculate the weighted average difference of net changes of body weight, body mass index (BMI), body fat percentage, fat mass, waist circumference, etc. Results: A total of 22 trials (870 overweight or obese participants) were reflected in the present meta-analysis. Analysis showed that soy products significantly reduced body weight, BMI, body fat percent and waist circumference in overweight or obese Asian populations (−0.37 kg, *P* = 0.010; −0.27 kg/m^2^, *P* = 0.042; −0.36%, *P* = 0.032; −0.35 cm, *P* = 0.049) and more significant effects were observed in non-menopausal women reduced body weight (−0.59 kg, *P* = 0.041), BMI (−0.59, *P* = 0.041) and waist circumference (−0.59 cm, *P* = 0.041) in overweight or obese populations. Conclusion: This meta-analysis showed that soy products have weight loss effects, mainly due to soy protein, isoflavone and soy fiber.

## 1. Introduction

Obesity is a major public health problem facing mankind today. The worldwide prevalence of obesity has increased more than doubled between 1980 and 2014 [1]. According to the World Health Organization [2], in 2016, there were 1.9 billion overweight adults worldwide, as well as 650 million obese patients. According to the latest data from the United States Centers for Disease Control and Prevention from 2015 to 2016, 39.8% of American adults and 18.5% of young people were obese [3]. In the United States alone, obesity-related medical costs have increased by $68.5 billion annually in the last 10 years [4]. If this trend continues, health care costs in the United States will reach $861 billion to $957 billion in 10 years, which appears to be astonishing [5]. In addition, in developing countries such as China, as the world’s most populous country, the incidence of overweight and obesity showed an alarming growth rate between 1991 and 2011, with obesity and central obesity increased by 8.1% and 32.0% respectively [6]. The Chinese government spends 2.46% of the country’s total medical expenditure on overweight, obesity and its complications every year, exceeding 24 billion yuan, with financial burden and the physical and psychological harm to people of obesity, namely the real risk, coming with it [7]. The Obesity Society (TOS) defines obesity as a disease, not only because it is a risk factor for many chronic diseases [8], but also because it is a serious condition that can weaken the body [9]. The most serious health problems brought by obesity are chronic non-communicable diseases such as hypertension, diabetes and myocardial infarction. For example, the risk of diabetes in obese people is 2.9 times that of ideal body weight, and the prevalence rate of diabetes in moderately obese people is 10 times that of ideal body weight. The prevalence of diabetes is 30 times higher in patients with over 135% body weight than these of ideal body weight. As for cardiovascular diseases, the prevalence of cardiovascular complications is as high as 64% obese and diabetic elderly patients alone [10]. In obese patients, the 30-day mortality rate after myocardial infarction is even as high as 16% [11]. In addition, obesity increases the risk of gallstones by nearly three times. The risk of some common tumors can be brought by obesity, such as colorectal cancer and adenocarcinoma [12,13]. As a debilitating condition, excessive fat accumulation can lead to physical and psychological problems, like gastrointestinal reflux, obstructive sleep apnea, anxiety and depression [8].

At present, the main methods of weight loss are surgery, medicine and so on, among which the healthiest and safest one is the combination of diet planning and exercise. There are many weight-loss programs that claim to be effective, but people have to stick to them, which is undoubtedly difficult in front of delicious food and they are not cheap. There are no recognized effective ways to lose weight, and some even pose health threats, such as the long-term effects of phentermine/topiramate on the heart and blood vessels, as well as diarrhea, nausea, vomiting, depression, anxiety and cognitive function caused by other drugs [14]. In addition, weight loss surgery not only increases the risk of metabolic disorders, cardiovascular disease and oncology diseases, but also has a certain impact on psychological function [15]. The results of a 2019 systematic review and meta-analysis show that weight loss surgery patients had higher self-harm or suicide attempt risk [16].

In recent years, the beneficial effect of soy products on body weight has aroused the interest in the scientists [17,18,19], whose biological effects in preventing metabolic disorders such as hyperlipidemia, cardiovascular disease and type 2 diabetes mellitus were commended [20]. Soy products herein refers to soy and a food processed from soy, and is mainly composed of soy protein, soy isoflavone and soy fiber, such as soy milk, soy shakes and so on. At present, it is believed that these beneficial effects may be attributed to high levels of protein, isoflavones, fibers and unsaturated fats [21]. However, the results of randomized controlled trials (RCTs) on the effects of soy products on overweight people are inconclusive. Some experiments suggested that soy products could help lose weight [22,23,24], while others could not [25,26]. Therefore, we collected data from RCTs and conducted a meta-analysis to explore the effect of soy products on the weight of overweight people.

## 2. Materials and Methods

### 2.1. Data Sources and Search Strategy

We searched PubMed (http://www.ncbi.nlm.nih.gov/pubmed/), Embase (http://www.embase.com/home) and Cochrane Library (http://www.cochranelibrary.com/), up to 31 January, 2019 for published studies in English to estimate RCTs that investigated the effect of soy products on weight in overweight population. We used a wide range of search terms, and the complete PubMed search strategy is presented in Table 1. In addition, we manually analyzed a reference list of original and recent reviews.

### 2.2. Criteria of Inclusion

Any study that met the following criteria was included: they (1) were restricted randomized controlled trials to investigate the association between intake of soy products and obesity-related anthropometric indicators; (2) had a target population of overweight or obese people; (3) reported a net change between the means of body weight, body mass index (BMI), body fat percentage, fat mass, fat-free mass, waist circumference, hip circumference or waist–hip ratio (WHR) before and after intervention with their corresponding standard deviations (SD), standard errors (SE), interquartile range (IQR), 95% confidence intervals (CI), or probability values; (4) provided the main ingredients and dosage of soy products. If two separate strata are included in one study, we see them as two trials.

Major reasons for exclusion were as follows: (1) lack of weight related data; (2) lack of an appropriate concurrent control group; (3) systematic differences between the experimental group and the control group.

### 2.3. Data Extraction and Quality Assessment

Two reviewers (MYZ and WBY) according to inclusion and exclusion criteria extracted relative information independently, any difference was resolved by discussion or adjudicated by the third reviewer (KTY). Information collected from each study included (1) the surname of the first author, publication year and country of origin; (2) the characteristics of the object (number, health status and mean age); (3) research design (single blind or double blind; cross over or parallel); (4) types and dosage of intervention, placebo and other treatment interventions; (5) assessment of the mean change in body weight, BMI, body fat percentage, fat mass, fat-free mass, waist circumference, hip circumference or WHR; (6) the methods of dietary assessment; (7) menopausal status ascertained.

### 2.4. Statistical Analysis

The net changes were determined as the difference between the baseline and final values. For original article that did not demonstrate the mean difference between the groups, we used each group independently reporting the reduction values in indicators to calculate it. We would set the control group’s mean change at zero and the treatment group’s mean change as the reported mean difference for the studies that provided only the changes in the variable mean value. We used 0.5 as the assumed correlation coefficient between the initial and final values when the SD of the mean differences was not reported. For data that uses SE and IQR, we used SD = SE/√(1/NE + 1/NC); (NE, and NC were the number of cases in the intervention group and the control group, respectively) and IQR ≈ 1.35 * SD for conversion.

The weighted mean difference (WMD) with 95% CI was used to represent the overall effect of the intervention by STATA 11.0 (Stata Corp.) and the statistical heterogeneity between studies were evaluated with Cochran’s Q test (*P* < 0.1). At the same time, the I^2^ statistic was tested and the heterogeneity was evaluated by the following indexes: I^2^= 75~100%, extreme heterogeneity; I^2^ = 50~75%, large heterogeneity; I^2^ = 25~50%, moderate heterogeneity and I^2^ = 0~25%, no heterogeneity. When the P of heterogeneity > 0.1, the fixed-effect model was utilized, otherwise, a random effect model was used. Evaluation of publication bias by Begg’s test and Egger’s test (*P* < 0.05 was considered statistically significant).

We conducted the subgroup analysis of each of the indicators. Subgroups were selected based on study characteristics and biological plausibility, including the type of countries and regions of the study population; menopausal status ascertained.

## 3. Results

### 3.1. Search Results

**Figure 1:**
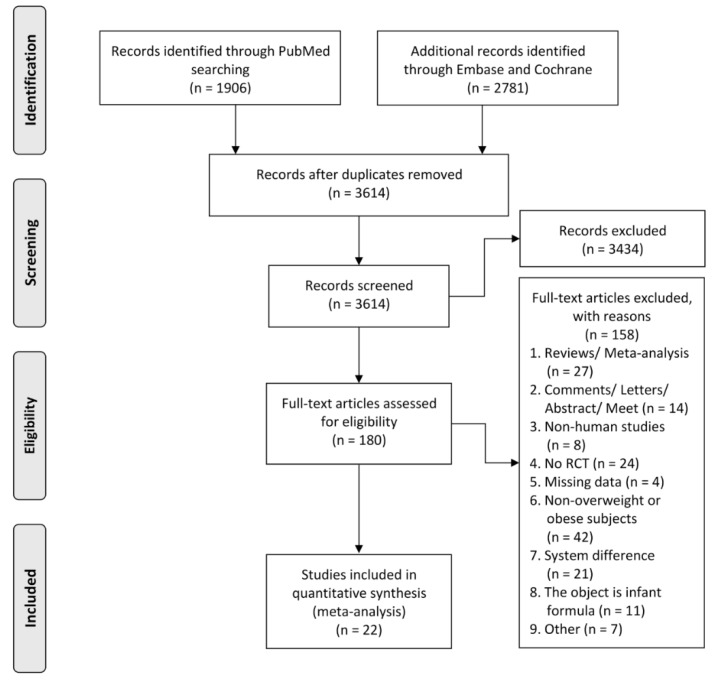
Flow diagram of study selection for meta-analysis.

According to our search strategy, 4687 articles were involved in, among which 180 provided detailed information. Finally, 22 of them were included in our current meta-analysis. The filtering process is shown in Figure 1.

### 3.2. Study Characteristics

The basic characteristics of the 22 studies involved are shown in Table 2, among which two were cross-designed and 20 were parallel designed. The sample number of the 22 trials ranged from 14 to 64, with a median of 39 and a total of 870 participants. Four studies were conducted in Iran, six in the United States and the rest in different countries. Of the 22 studies, 11 included both men and women, and 10 focused on women, including five postmenopausal and three non-menopausal women. All trials were conducted in overweight or obese people, except four of them were conducted only in obese people. BMI was used to determine overweight or obesity status in 18 trials. The minimum requirement for inclusion was that BMI ≥ 23 and six studies also set limited waist circumference or WHR. In three studies, the percentage of fat mass percentage (FM%) was used to define obesity (FM% > 40% and FM% > 35%) and only two studies did not report the method for determining overweight or obesity status. The duration of intervention in these studies ranged from two to 24 weeks, with soy protein, soy isoflavone, soy fiber and some soy products with regional characteristics being the main intervention products. The average daily intervention dose was 25.5 g for soy protein, 60–135 mg for isoflavone and 240–720 mL for soy milk. All control groups were given non-soy products. The control groups in these studies were usually given whey protein or placebo. In one study, simple resistance training was selected as the control group, and the soy products in the intervention group were compared with the resistance training.

### 3.3. Effects of Soy Products on Body Weight

**Figure 2:**
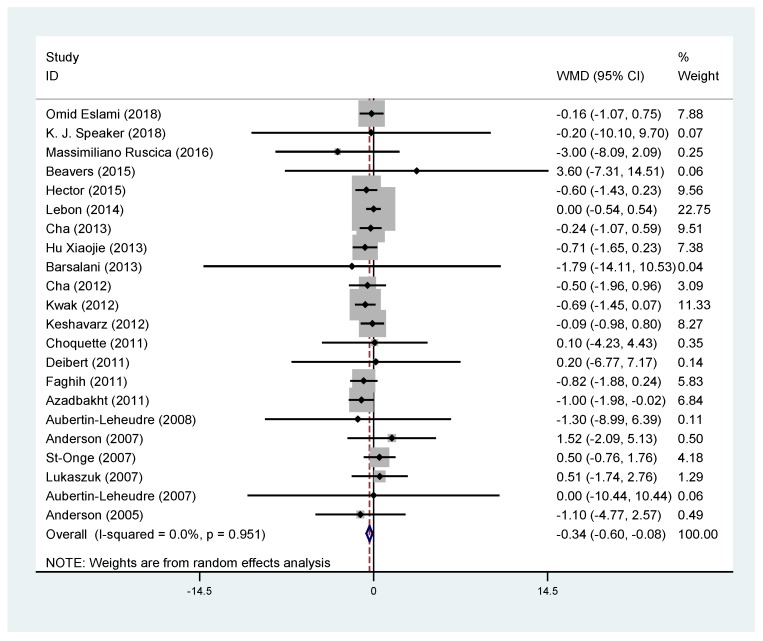
Meta-analysis of the effect of soy products on body weight. ID: identification; WMD: weighted mean difference.

**Figure 3:**
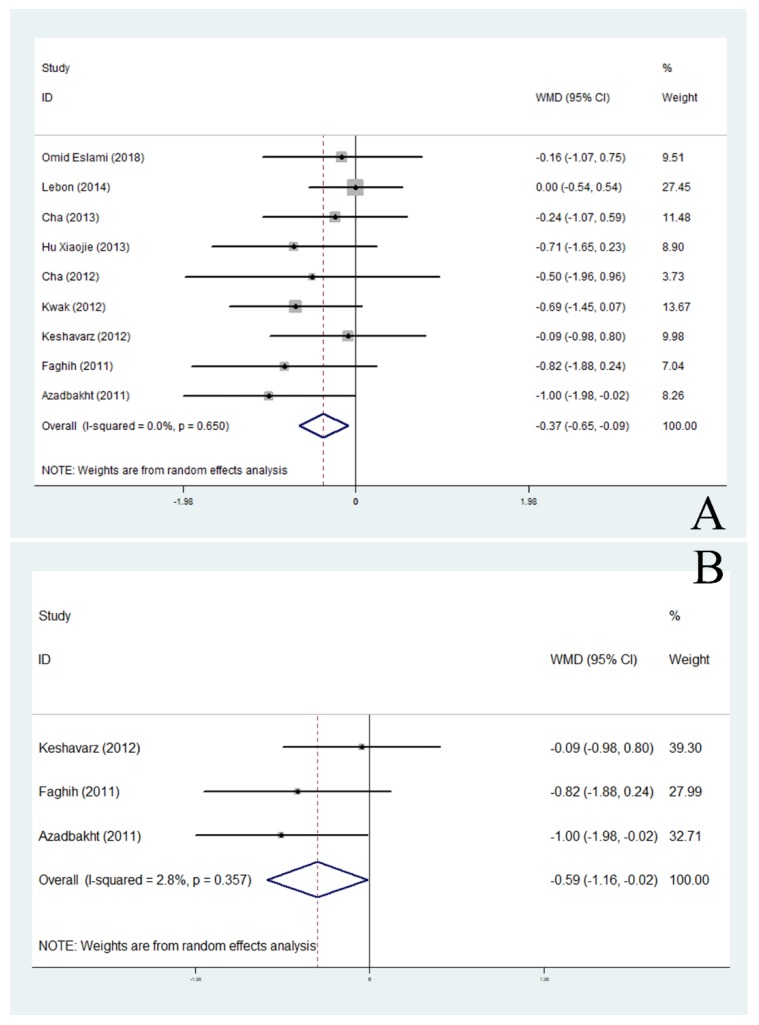
Meta-analysis of the effect of soy products on body weight in Asian population (**A**) and non-menopausal (**B**). ID: identification; WMD: weighted mean difference.

Twenty-two RCTs met our inclusion criteria and a total of 469 soy products supplement subjects and 448 control subjects were identified (Table 3). Soy products had a significant overall effect on body weight without heterogeneity (WMD, −0.34; 95% CI: −0.60 to −0.08; *P* = 0.009) (Figure 2). Subgroup analysis showed that soy products significantly reduced the weight of premenopausal obese women (*P* < 0.05) and obese people in Asian countries (*P* < 0.05). In addition, soy products showed significant weight loss effects in both male and female subjects (*P* < 0.05) and in studies with more than 39 samples (*P* < 0.05) (Figure 3).

### 3.4. Effects of Soy Products on BMI

**Figure 4:**
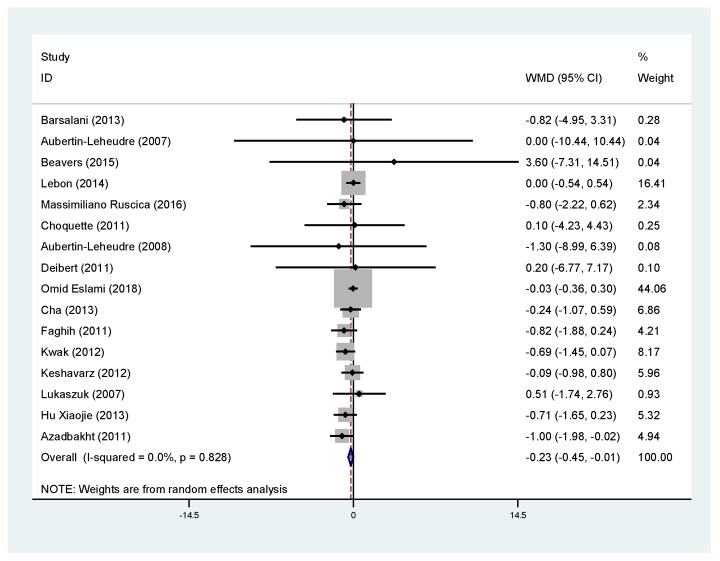
Meta-analysis of the effect of soy products on body mass index (BMI). ID: identification; WMD: weighted mean difference.

**Figure 5:**
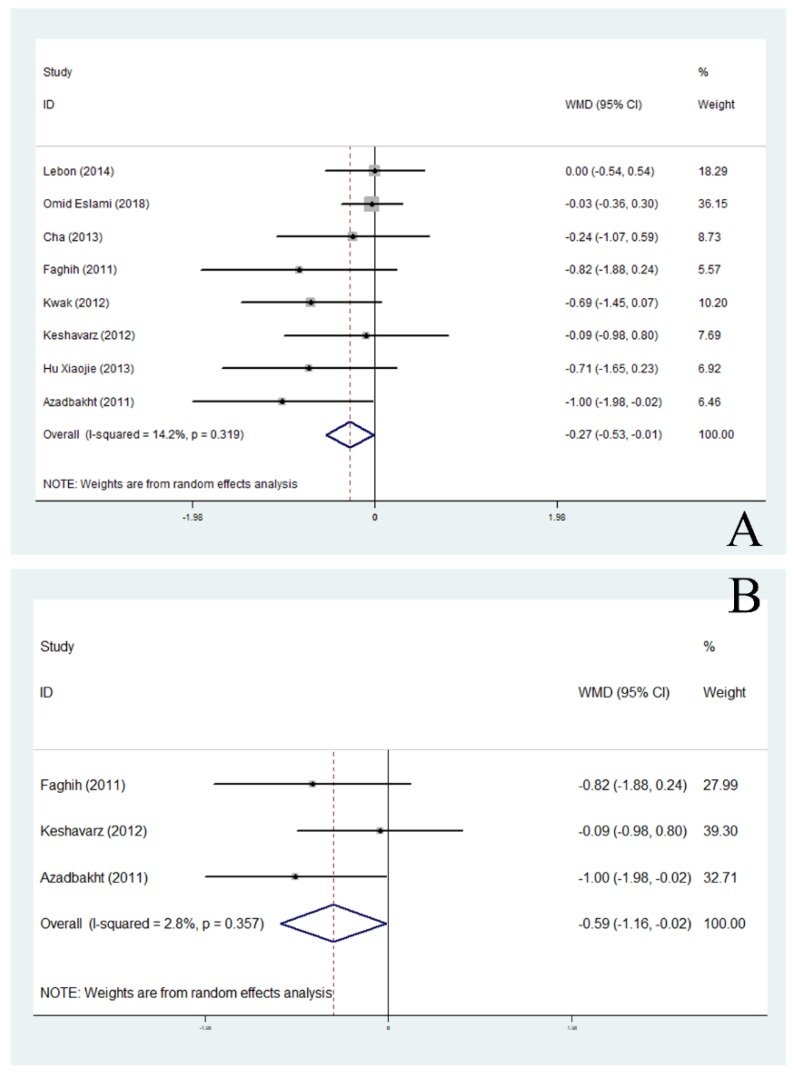
Meta-analysis of the effect of soy products on BMI in the Asian population (**A**) and non-menopausal women (**B**). ID: identification; WMD: weighted mean difference.

Sixteen trials on the relationship between supplementary soy products and BMI met our inclusion criteria (Table 3). In this analysis, 329 people took soy products and 318 people took placebo. Overall, BMI of obese people who consumed soy products after the study was significantly lower than that of the control group (WMD, −0.23; 95% CI: −0.45 to −0.01; *P* = 0.040) (Figure 4). Meanwhile, subgroup analysis showed that soy products significantly reduced BMI of non-climacteric overweight or obese women (*P* < 0.05) and Asian overweight or obese people (*P* < 0.05) (Figure 5).

### 3.5. Effects of Soy Products on Body Fat Percentage and Fat Mass

Twelve studies on percentage of fat mass (E: 248, C: 232) and 16 studies on body fat weight (E: 310, C: 297) were included in this study (Table 3). Compared with control group, fat mass in experimental group was significantly lower (WMD, −0.32; 95% CI: −0.61 to −0.03; *P* = 0.031). The number of overweight or obese people in Asian countries showed similarly significant declines in the percentage of body fat and fat mass (*P* < 0.05). What’s worth mentioning is that in studies involving both men and women, fat mass also showed a significant decrease compared with the control group (*P* < 0.05).

### 3.6. Effects of Soy Products on Waist Circumference and Hip Circumference

Overall, no significant changes in waist circumference (E: 321, C: 307, WMD, −0.29; 95% CI: −0.62 to 0.04; *P* = 0.083), hip circumference (E: 121, C: 116, WMD, −0.39; 95% CI: −0.85 to 0.07; *P* = 0.099) and WHR (E: 123, C: 123, WMD, −0.00; 95% CI: −0.04 to 0.04; *P* = 0.02) were observed (Table 3). Subgroup analysis showed soy products supplements had a significant effect on decreasing waist circumference of non-menopausal obese women (*P* < 0.05) and Asian obese people (*P* < 0.05).

### 3.7. Publication Bias

**Figure 6:**
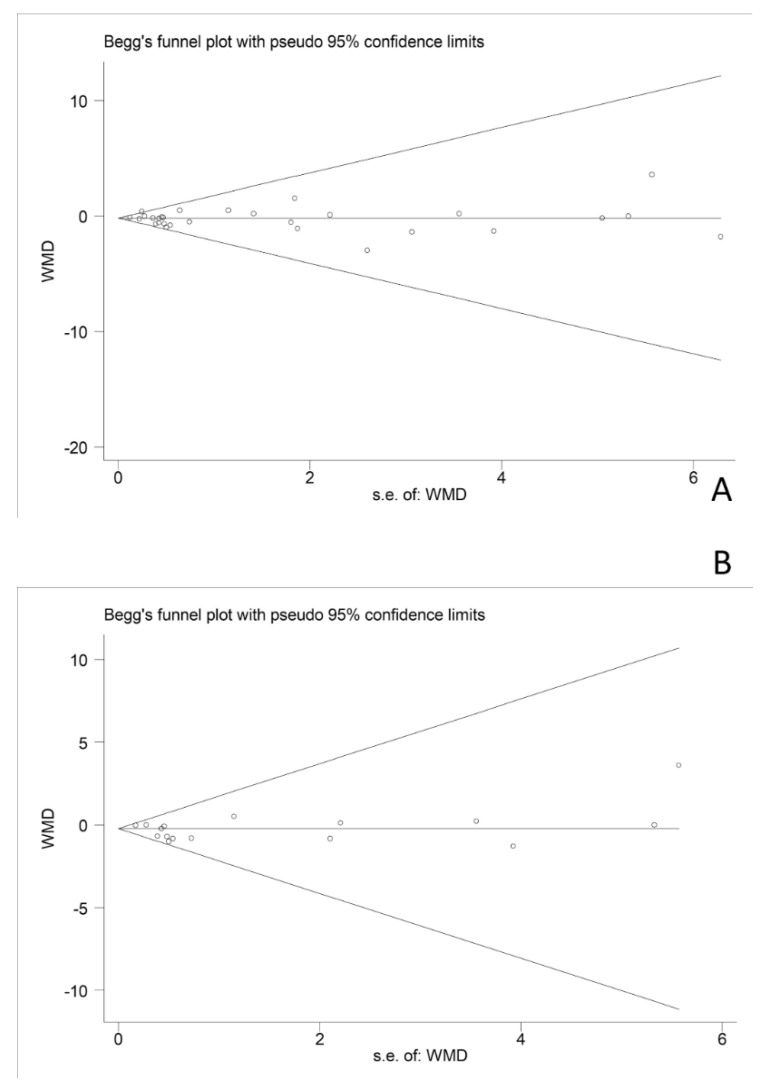
Publication bias funnel chart of the effects of soy products on body weight (**A**) and BMI (**B**) in overweight or obese people.

No indicators showed any bias when examining funnel maps while the results from Begg’s tests and Egger’s also showed no evidence of publication bias. Take the publication bias funnel of body mass and body mass index as an example (Figure 6).

## 4. Discussion

This meta-analysis including 22 trials showed that soy products reduced body weight by 0.34 kg, BMI by 0.23 kg/m^2^ and fat mass by 0.32 kg in overweight or obese people. Subgroup analysis showed that soy products significantly reduced body weight, BMI, the percentage of body mass, fat mass and waist circumference in overweight or obese Asian people, and they can also reduce body weight, BMI and waist circumference of overweight or obese non-menopausal women.

The highlight of the present research is that intervention trials by using various types of soy products such as soy protein, isoflavones, soy milk, soy shakes and some other soy products with regional characteristics were included. This makes our research more instructive. A recently published meta-analysis in 2017 by Masoumeh Akhlaghi et al. reported that no statistically significant overall effect of soy on weight, waist circumference, or fat mass [41]. But in this study, the subjects were adults aged 18 and older, and the interventions were only soybeans and soy isoflavones. In contrast, our meta-analysis looked at overweight or obese people, and we included a wider range of interventions. Meanwhile, we studied the areas where the study population was located and found that soy products had a positive impact on overweight or obese people in Asia by subgroup analysis.

As for the analysis of data from World Health Survey in 70 countries with different income from 2002 to 2004, which is carried out by Mohd Masood, it was claimed that BMI in most high-income countries was higher than the average of 25.0, while that in low-income countries was lower than 25.0 [42]. This can be explained by the fact that the BMI of people in developed countries is generally higher than that in developing countries such as these in Asia, while the control of food intake by obese individuals is poorer than that of those who with ideal body weight [43,44]. Therefore, people in developing countries can reduce their energy intake by consuming soy products while these in developed countries are unlikely to do so. Moreover, the animal-based dietary patterns in developed countries are different from the plant-based one in developing countries. On the one hand, it is feasible to use vegetable foods such as soy products to replace the intake of certain other foods among the Asian people, who are accustomed to vegetable foods. On the other hand, it is difficult for people in European and US, who mainly eat animal-based food, to change their animal-based dietary habit to the plant-based one, which makes this argument more convincing.

It is worth mentioning that since Frayling first proposed in 2007 that fat mass and obesity associated (FTO) gene is associated with obesity [45], a large number of studies have been carried out around the world to confirm the relationship between FTO gene and BMI [46,47,48,49]. However, there are few studies on the differences of FTO gene polymorphism in different countries and regions. But the current research on “thrifty genotype” seems to be the explanation to this phenomenon, which hypothesizes that when food is abundant, genetic genes promote fat accumulation, thus conducive to the hunting and gathering of human ancestors [50]. In a study of the spatial distribution of thrift genes, the angiotensin-converting enzyme (ACE) gene without 287-bp AluYa5 element (D) was used to replace the thrift genes [51]. The frequency of D allele was the lowest in Central Asia, but higher in Europe and America. Consequently, the expression of thrift genes in Europeans and Americans may be higher than that in Asians. In 13 of the studies we included, the interventions were soy protein, soy fiber and protein-rich soy milk, which produced a sense of satiety. After eating these foods, people may reduce their intake of other foods because of a sense of satiety. Furthermore, thrift genes promote the accumulation of fat. Thus, it is reasonable to believe that when soy products are used instead of certain other foods, Asians are more likely to lose weight, while people in Europe and the United States are more likely to increase food utilization and the storing calories by thrift genes, so weight loss is not significant.

More significant effects were observed in non-menopausal women that soy products supplementation significantly reduced body weight, BMI and waist circumference of premenopausal women, while no similar results were observed in postmenopausal women. It is reported that the transcripts of fatty acid transporters, peroxisome proliferator-activated receptor γ and coded adiponectin in hip fat of postmenopausal women are significantly higher than those of pre-menopausal women and postmenopausal women treated with hormones. On the contrary, the transcripts of hormone sensitive lipase, long chain acyl CoA dehydrogenase and transcripts for acetyl CoA carboxylase alpha in abdominal fat of premenopausal women were significantly higher than those of postmenopausal women and postmenopausal women after hormone therapy. Therefore, changes in weight gain and fat metabolism during menopause may lead to fat accumulation, which may lead to differences in body composition and muscle tissue content between postmenopausal women and pre-menopausal women [52,53]. A randomized cross-sectional study in 2017 found a significant negative correlation between body fat mass and stride length (an indicator of postural stability) in postmenopausal women [54]. Among the obese women involved in our study, this can be one of the reasons for the decline in exercise capacity and volume. The increase of weight and fat content among postmenopausal women, as well as the decline in exercise capacity, may be the reason that soy products supplements cannot significantly reduce postmenopausal women’s obesity-related anthropometric indicators. In contrast to our findings, a meta-analysis of the relationship between isoflavones supplementation and body weight in non-Asian postmenopausal women published in 2013 showed that soy isoflavones supplementation significantly reduced body weight [−0.52 kg (95% CI: −0.89 to −0.134)] [55]. Compared with this study, several reasons may lead to the different results. Firstly, as we mentioned earlier, our research covers a wider range of soy products, not just soy isoflavones. Secondly, only non-Asian women got involved in their study. In the current meta-analysis, we studied all obese people and both premenopausal and postmenopausal women were covered in the subgroup analysis, which including four trials on non-Asian people (Canada) and four trials on Asian people. Overall, soy products may be beneficial to lose weight, and these findings are supported with experimental data.

The weight loss effect of soy products can be explained from three aspects: soy protein, soy isoflavones and soy fiber. Proteins can reduce body weight by increasing fullness and regulating mTOR signaling pathways. Natural soy protein can bind to soy isoflavones firmly and protect isoflavone from degradation after heat treatment [56].

As for soy isoflavones, the weight loss effect of it was emphasized by many people and studies [19]. Previous studies have shown that soy isoflavones can reduce fat accumulation by inhibiting fat production and increasing FA b-oxidation [57]. In addition, it is reported by some studies that soy isoflavone taken by mouth can improve insulin resistance [58,59,60], that is closely related to obesity and long-term-low-dose isoflavone supplementation has significant effect on controlling blood glucose [19,61,62,63,64]. It is well known that estrogen can control food intake, regulate energy consumption and prevent fat from being accumulation [65]. At the same time, isoflavones, are classified as phytoestrogens precisely because they are structurally similar to 17-b-estradiol, allowing them to be bound to estrogen receptors and mimic its activity [66]. Therefore, the anti-obesity effect of isoflavones can also be achieved by binding with estrogen receptors. Some studies reported that isoflavones played an important role in lipid metabolism by regulating PPAR-regulated genes and transcription factors such as the sterol regulatory element binding protein (SREBP) [67,68], which were closely related to the metabolism of glucose and fatty acids. [68,69,70,71]. In addition, isoflavones could affect stearoyl coenzyme A desaturase 1, a key enzyme in obesity [72].

As the main ingredient of legumes, dietary fiber can promote satiety, thus reducing the self-intake of food [73,74,75,76,77,78]. More and more studies have shown that eating high-fiber foods or whole grains can effectively control body weight by delaying carbohydrate absorption [73,79,80,81,82,83]. In a prospective cohort studies, women who had dietary habits of high fiber or whole grains were half as likely to be obese within 12 years as other women [80]. Therefore, higher dietary fiber content in soy products may contribute to its weight loss effect.

Our meta-analysis has many strengths. Firstly, due to the frequent consumption of soy and soy products, we have included all the soy products studies in the meta-analysis, with the results being more practical and generalized. Secondly, the anthropometric indicators related to obesity are comprehensive. Thirdly, a series of subgroup analysis were conducted to determine the factors affecting the results. Most of the subgroup had a heterogeneity of zero, while the others had a low heterogeneity. Finally, there is no evidence of publication bias existed in this meta-analysis by Egger’s test and the funnel diagram.

Some limitations need to be considered when interpreting the results of this meta-analysis. Firstly, it is impossible to analyze the interaction between each soy products and body weight due to the limitation of the size and quantity of the experiment. Secondly, due to the insufficient number of related studies in the indicators, it is impossible to evaluate the dose-effect relationship. Therefore, the real effect need to be demonstrated in well-designed, larger randomized controlled trials for overweight or obese subjects.

## 5. Conclusions

Overall, this meta-analysis showed that soy products supplementation could be helpful in reducing body weight, BMI, body fat percentage, fat mass and waist circumference in overweight or obese Asians. Soy products have significant control over body weight, BMI and waist circumference of premenopausal overweight or obese women. Being a risk factor for diabetes, hypertension and cancer, obesity is known for its harmful effects on human health [37]. Our meta-analysis shows that soy products can reduce the weight of obese people in Asia and obese women who are not suffering from menopause, which may help maintain the health of these people. The randomized controlled trial of higher quality RCTs is expected to compare the effects of various soy products on obesity-related variables and to explore the relationship and the possible mechanisms.

## Figures and Tables

**Table 1:** The search strategy in PubMed of soy products and weight.

Search Terms	
1	Soy Milk[Mesh] OR Soybean Oil[Mesh] OR Soybean Proteins[Mesh] OR Soybeans[Mesh] OR Soy Foods[Mesh] OR Isoflavones[Mesh]
2	soy[Title/Abstract] OR soybean*[Title/Abstract] OR legume*[Title/Abstract] OR soya[Title/Abstract] OR soybean oil[Title/Abstract] OR soybean protein[Title/Abstract] OR isoflavone*[Title/Abstract] OR phytoestrogens[Title/Abstract] OR genistein[Title/Abstract] OR daidzein[Title/Abstract] OR soy milk[Title/Abstract] OR tofu[Title/Abstract] OR miso[Title/Abstract] OR natto[Title/Abstract]
3	1 OR 2
4	Body Weight[Mesh] OR Weight Loss[Mesh] OR Weight Gain[Mesh] OR Body Weight Changes[Mesh] OR Body Mass Index[Mesh] OR Obesity[Mesh] OR Overweight[Mesh]
5	body mass index[Title/Abstract] OR fatness[Title/Abstract] OR body fatness[Title/Abstract] OR weight change[Title/Abstract] OR weight variability[Title/Abstract] OR weight gain[Title/Abstract] OR weight loss[Title/Abstract] OR obesity[Title/Abstract] OR overweight[Title/Abstract] OR body weight[Title/Abstract] OR adiposity[Title/Abstract] OR fat mass[Title/Abstract] OR body fat[Title/Abstract] OR body size[Title/Abstract] OR body composition[Title/Abstract] OR central obesity[Title/Abstract]
6	4 OR 5
7	Humans[Mesh]
8	3 AND 6 AND 7

“*”: wildcard character, which can represent 0-n characters.

**Table 2:** Characteristics of all trials included in the present meta-analysis.

Author, Year, (Reference) Country	Design	Sample Size, Mean Age, Subject, Duration (Week)	Dietary Assessment	Experimental Group Type	Control Group Type	Index
Speaker, 2018 America [18]	NB, P	57, 42.00, BMI 27~40 people, 4	NR	Soy protein	No-soy protein diet	a; b; c; d; e;
Eslami, 2018 Iran [27]	NB, P	64, 45.70 Overweight or obese adults with nonalcoholic fatty liver disease, 8	NR	Soy milk	No-soy milk diet	a; b; f;
Ruscica, 2016 Italy [28]	NB, P	53, 58.90 Overweight or obese males and postmenopausal women, 12	NR	Soy protein	/	a; b; f; g; h
Hector, 2015, Canada [29]	DB, P	28, 52.00, BMI 28~50, nondiabetic, 2	Three-day food record	Soy protein	Whey	a; d; e;
Beavers, 2015, America [30]	SB; P	24, 68.40 ± 5.50 Older and abdominally obese adults, 12	Keep a log of all foods consumed	Isoflavones	Whey and egg proteins	a; b; c; d; e; f; g;
Lebon, 2014, Canada [31]	DB; P	30, 59.50 ± 4.50 Overweight or obese women, 24	NR	Isoflavone + exercise	Placebo + exercise/four capsules	a; b; c; d; f; g;
Hu, 2013, China [32]	NR; P	39, 23.17 Overweight and obese college adults, 12	Self-administered dietary questionnaire	Soy fiber + biscuit	Biscuit	a; b; c; d; f; g;
Cha, 2013, South Korea [33]	DB; P	53, 42.55 Overweight adults, 12	Three-day food record	Kochujang (KCJ) supplement	Placebo supplement	a; b; c; d; h;
Barsalani, 2013, Canada [34]	DB; P	39, NR Overweight women, 24	Three-day food record	Isoflavone + exercise	Placebo + exercise	a; b; d; e; f;
Kwak, 2012, South Korea [22]	DB; P	64, 37.73 Overweight or obese people, 12	24h recall and a semi-quantitative food frequency questionnaire	Black soy peptide	Casein	a; b; c; d;
Keshavarz, 2012, Iran [25]	NB; X	24, 37.70 ± 1.30 Overweight or obese women, 4	Three-day food record	Soy milk	Cow’s milk	a; b; f; h;
Cha, 2012, South Korea [24]	DB; P	51, 39.87 Overweight adults, 12	Three-day food record	Freeze-dried Doenjang	Inactive ingredients	a; c; d; h;
Faghih, 2011, Iran [23]	NB; P	41, 37.88 Overweight or obese women, 8	24-h dietary records	Soy milk	A 500 kcal/day deficit diet	a; b; c; d; f; h;
Deibert, 2011, Germany [35]	NB, P	26, 55.70 ± 4.10 Moderately overweight middle aged males, 12	Self-reported records	Soy–yogurt–honey + the resistance training	The resistance training	a; b; d; e; f;
Choquette, 2011, Canada [36]	DB; P	45, 58.49 Overweight or obese women, 24	Three-day food record	Isoflavones	Placebo capsules	a; b; c; d; e; f; g;
Azadbakht, 2011, Iran [37]	NB; X	23, 22.08 ± 2.71 Overweight and obese female youths, 6	Three-day food record	Soy milk	Cow’s milk	a; b; f; g;
Aubertin-Leheudre, 2008, Canada [26]	DB, P	39, 57.40 Obese women, 24	Three-day food record	Isoflavones	Placebo capsules	a; b; c; d; e; f;
St-Onge, 2007, America [38]	NR, P	47, 39.04 Overweight women, 12	Three-day food record	Soy protein	/	a; f;
Lukaszuk, 2007, America [17]	NB, P	14, 31.57 Overweight or obese adults, 8	Three-day food record	Soy milk	Skim milk	a; b; c; d; e;
Aubertin-Leheudre, 2007, Canada [19]	DB; P	22, NR Obese women, 24	NR	Isoflavones	Placebo capsules	a; b; c; d;
Anderson, 2007, America [39]	SB; P	35, 45.20 Obese women, 16	Keep daily records	Soy shakes	Casein shakes	a; d; e; f;
Anderson, 2005, America [40]	NB, P	52, 47.40 Overweight or obese adults, 12	Lifestyle dairy	Soy-based meal replacement	Milk-based meal replacement	a; f;

NR: no report; P: parallel control study; SB: single blind; DB: double blind; NB: non-blind; X: cross-over. a: body weight (kg); b: BMI (kg/m^2^); c: body fat percentage (%); d: fat mass (kg); e: fat-free mass (kg); f: waist circumference (cm); g: hip circumference (cm); h: waist–hip ratio (WHR); “+”: and.

**Table 3:** Pooled estimates of treatment effects on obesity-related anthropometric measures in subgroups of trials.

Indicators	Subgroups	No. of Studies	Net Change	95% CI	*p* Value	I^2^ (%)
Body weight (kg)	All trials	22	−0.34	−0.60, −0.08	0.009 *	0.0
	Area					
	Asia	9	−0.37	−0.65, −0.09	0.010 *	0.0
	North America	11	−0.17	−0.79, 0.46	0.606	0.0
	Menopausal status					
	Post-menopausal	5	−0.01	−0.54, 0.52	0.976	0.0
	Non-menopausal	3	−0.59	−1.16, −0.02	0.041 *	2.8
	Sample					
	>Median	10	−0.39	−0.78, −0.00	0.049	0.0
	≤Median	12	−0.30	−0.64, 0.04	0.083	0.0
	Blind method					
	DB	9	−0.31	−0.65, 0.03	0.075	0.0
	SB	2	1.73	−1.70, 5.16	0.324	0.0
BMI (kg/m^2^)	All trials	16	−0.23	−0.45, −0.01	0.040 *	0.0
	Area					
	Asia	8	−0.27	−0.53, −0.01	0.042 *	14.2
	North America	6	0.19	−1.51, 1.90	0.826	0.0
	Menopausal status					
	Post-menopausal	5	−0.02	−0.55, 0.51	0.947	0.0
	Non-menopausal	3	−0.59	−1.16, −0.02	0.041 *	2.8
	Sample					
	>Median	6	−0.21	−0.48, 0.06	0.463	0.0
	≤Median	10	0.00	−10.44, 10.44	0.818	0.0
	Blind method					
	DB	7	−0.24	−0.62, 0.15	0.228	0.0
	SB	1	3.60	−7.31, 14.51	0.518	-
Body fat percentage (%)	All trials	12	−0.23	−0.56, 0.09	0.154	0.0
	Area					
	Asia	6	−0.36	−0.69, −0.03	0.032 *	0.0
	North America	6	0.71	−0.16, 1.58	0.108	0.0
	Menopausal status					
	Post-menopausal	4	−0.01	−0.54, 0.53	0.986	0.0
	Non-menopausal	1	−0.82	−1.88, 0.24	0.130	-
	Sample					
	>Median	4	−0.16	−0.83, 0.50	0.630	0.0
	≤Median	8	−0.25	−0.66, 0.17	0.250	0.0
	Blind method					
	DB	7	−0.25	−0.62, 0.12	0.191	0.0
	SB	1	3.60	−7.31, 14.51	0.518	-
Fat mass (kg)	All trials	16	−0.32	−0.61, −0.03	0.031 *	0.0
	Area					
	Asia	6	−0.36	−0.69, −0.03	0.032 *	0.0
	North America	9	−0.18	−0.80, 0.44	0.566	0.0
	Menopausal status					
	Post-menopausal	5	−0.02	−0.55, 0.51	0.947	0.0
	Non-menopausal	1	−0.82	−1.88, 0.24	0.130	-
	Sample					
	>Median	5	−0.35	−0.83, 0.13	0.149	0.0
	≤Median	11	−0.30	−0.67, 0.07	0.109	0.0
	Blind method					
	DB	9	−0.31	−0.65, 0.03	0.072	0.0
	SB	2	1.73	−1.70, 5.16	0.324	0.0
Waist circumference (cm)	All trials	15	−0.29	−0.62, 0.04	0.083	0.0
	Area					
	Asia	6	−0.35	−0.70, −0.00	0.049 *	0.0
	North America	7	0.34	−0.70, 1.39	0.517	0.0
	Menopausal status					
	Post-menopausal	4	−0.02	−0.55, 0.51	0.946	0.0
	Non-menopausal	3	−0.59	−1.16, −0.02	0.041 *	2.8
	Sample					
	>Median	6	−0.38	−1.05, 0.30	0.275	0.0
	≤Median	9	−0.27	−0.64, 0.11	0.168	0.0
	Blind method					
	DB	4	−0.02	−0.55, 0.51	0.946	0.0
	SB	2	1.73	−1.70, 5.16	0.324	0.0
Hip circum-ference (cm)	All trials	6	−0.39	−0.85, 0.07	0.099	6.1
	Area					
	Asia	3	−0.46	−1.10, 0.18	0.161	48.2
	North America	2	0.58	−3.45, 4.60	0.779	0.0
	Menopausal status					
	Post-menopausal	2	0.00	−0.53, 0.54	0.996	0.0
	Non-menopausal	1	−1.00	−1.98, −0.02	0.046	-
	Sample					
	>Median	2	−1.19	−3.65, 1.27	0.343	-
	≤Median	4	−0.43	−1.02, 0.17	0.157	31.1
	Blind method					
	DB	2	0.00	−0.53, 0.54	0.996	0.0
	SB	1	3.60	−7.31, 14.51	0.518	-

“-”: there is only one study in this subgroup and there is no way to compare heterogeneity. *: *P* < 0.05, there was statistical difference.
